# Supplementary material for: A spectrum of modularity in multi‐functional gene circuits
Source: Mol Syst Biol. 2017 Apr 1;13(4):925. doi: 10.15252/msb.20167347 (PMC5408781; doi:10.15252/msb.20167347)
Supplement: Supplementary file 1 — Appendix [file MSB-13-925-s001.pdf]

Appendix

Contents

List of Figures

S1 Noise can drive lateral inhibition . . . 2
S2 Minimal induction and inhibition circuits classified into dynamical mechanisms . . . 3
S3 Distinct mechanisms make use of distinct regulatory logics . . . 5
S4 Specialization of bi-functional topologies . . . 9
S5 Complete atlas of multi-functional gene circuits . . . 10
S6 Modular candidates for multi-functionality . . . 11
S7 Function-switching mechanism of Pattern-Convertor . . . 12
S8 Phase portrait analysis of induction and inhibition minimal circuits . . . 14
S9 Bifurcation as a function-switching mechanism . . . 15

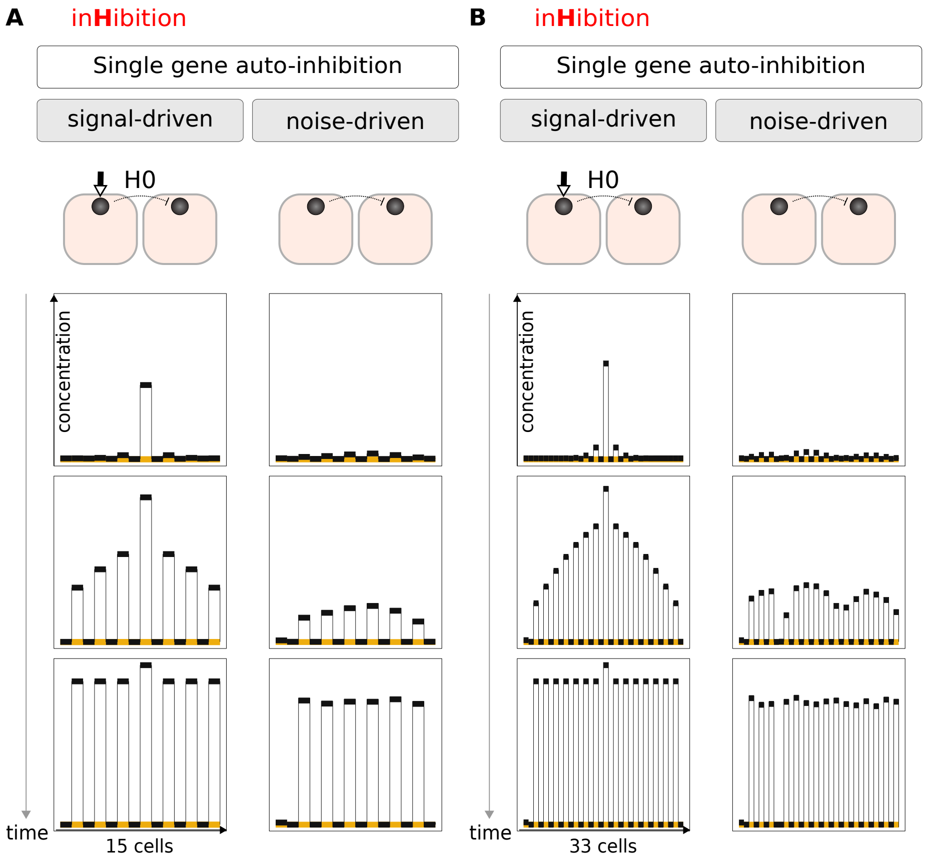

Appendix Figure S1: **Noise can drive lateral inhibition.** The presence of the trigger signal T at the center of the tissue is not needed in order to obtain a fine-grained pattern. Indeed, noise helps initial asymmetries self-amplify and can drive the formation of a fine-grained pattern [2, 1]. We show how the same gene circuit can achieve inhibition with or without an initial trigger. We show simulations with distinct numbers of cells (15 (A) or 33 (B)). It has also been suggested that noise has a beneficial role in refining lateral inhibition patterns [1].

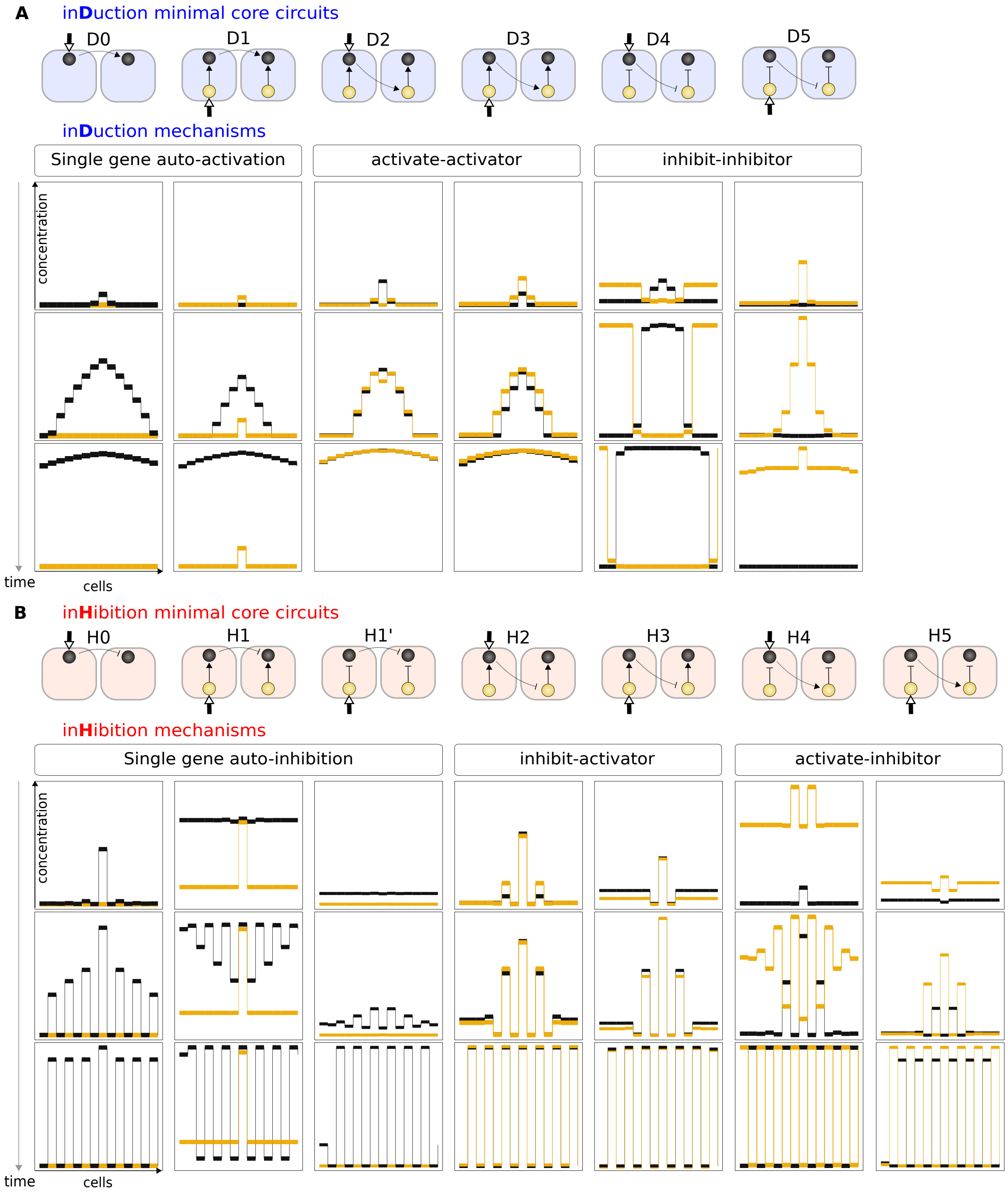

Appendix Figure S2: **Minimal induction and inhibition circuits classified into dynamical mechanisms.** Within a given mechanism we find at least two equivalent circuits, depending on which gene receives the trigger signal T. (A) *Auto-activation* leads to an independent expansion of the signaling gene. *Activate-activator* drives an in phase expansion of both genes. *Inhibit-inhibitor* leads to an out of phase expansion as the signaling gene 'pushes away' the cell-autonomous gene. (B) Inter-cellular *auto-inhibition* of the signaling gene causes the neighbor cell to lose its inhibitory potential on next cell, which in turn adopts a constitutive high concentration. In *inhibit-activator* single cells adopt high expression levels for both genes. Last, in *activate-inhibitor* intra-cellular inhibition causes single cells to adopt opposite concentration levels for black and yellow gene. The parameter sets used for each simulation $[w_1;w_5;\alpha_A;\alpha_D]$ correspond to the strength of inter-cellular (w_1) and intra-cellular (w_5) interactions (see labels in Fig 2B) and α parameters of both genes: D0=[1.02;0;0;7.56], D1=[1.84;1.36;6.68;15.01], D2=[2.67;2.80;26.46;6.30], D3=[5.11;1.25;6.43;22.10], D4=[-6.38;-4.95;-26.15;-18.16], D5=[-8.87;-0.12;-1.10;4.74], H0=[-8.86;0;0;-1.67], H1=[-1.8;8.41;1.88;-8], H1'=[-1.31;-0.75;11.44;-10.15], H2=[-8.08;1.19;-8.78;15.70], H3=[-8.30;3.64;-8.25;15.15], H4=[4.20;-0.15;5.07;-5.62], H5=[2.40;-5.58;7.99;2.57].

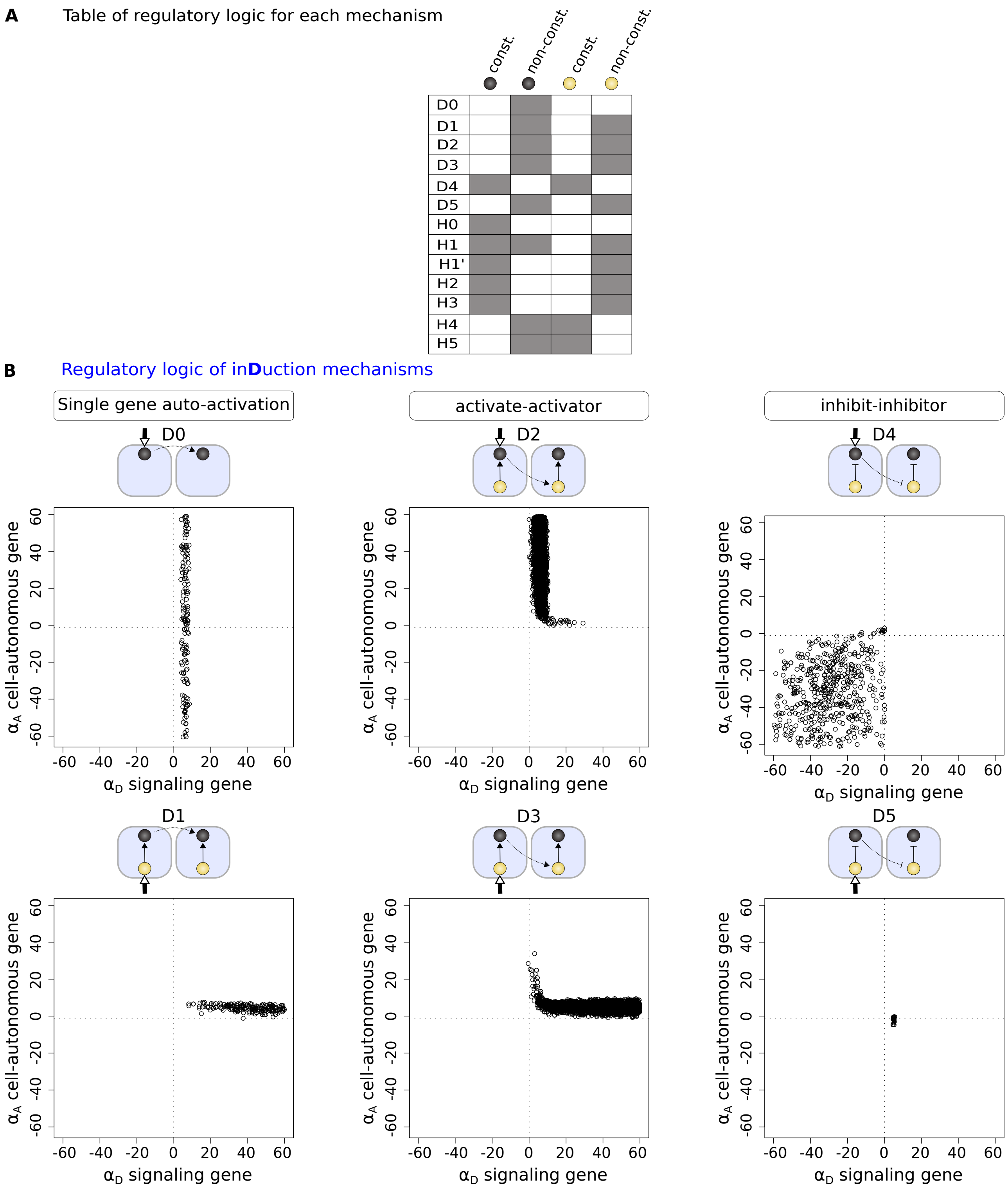
A
Table of regulatory logic for each mechanism
const.
non-const.
const.
non-const.
D0
D1
D2
D3
D4
D5
H0
H1
H1'
H2
H3
H4
H5
B
Regulatory logic of inDuction mechanisms
Single gene auto-activation
activate-activator
inhibit-inhibitor
D0
D2
D4
D1
D3
D5
αA cell-autonomous gene
αD signaling gene
-60
-40
-20
0
20
40
60

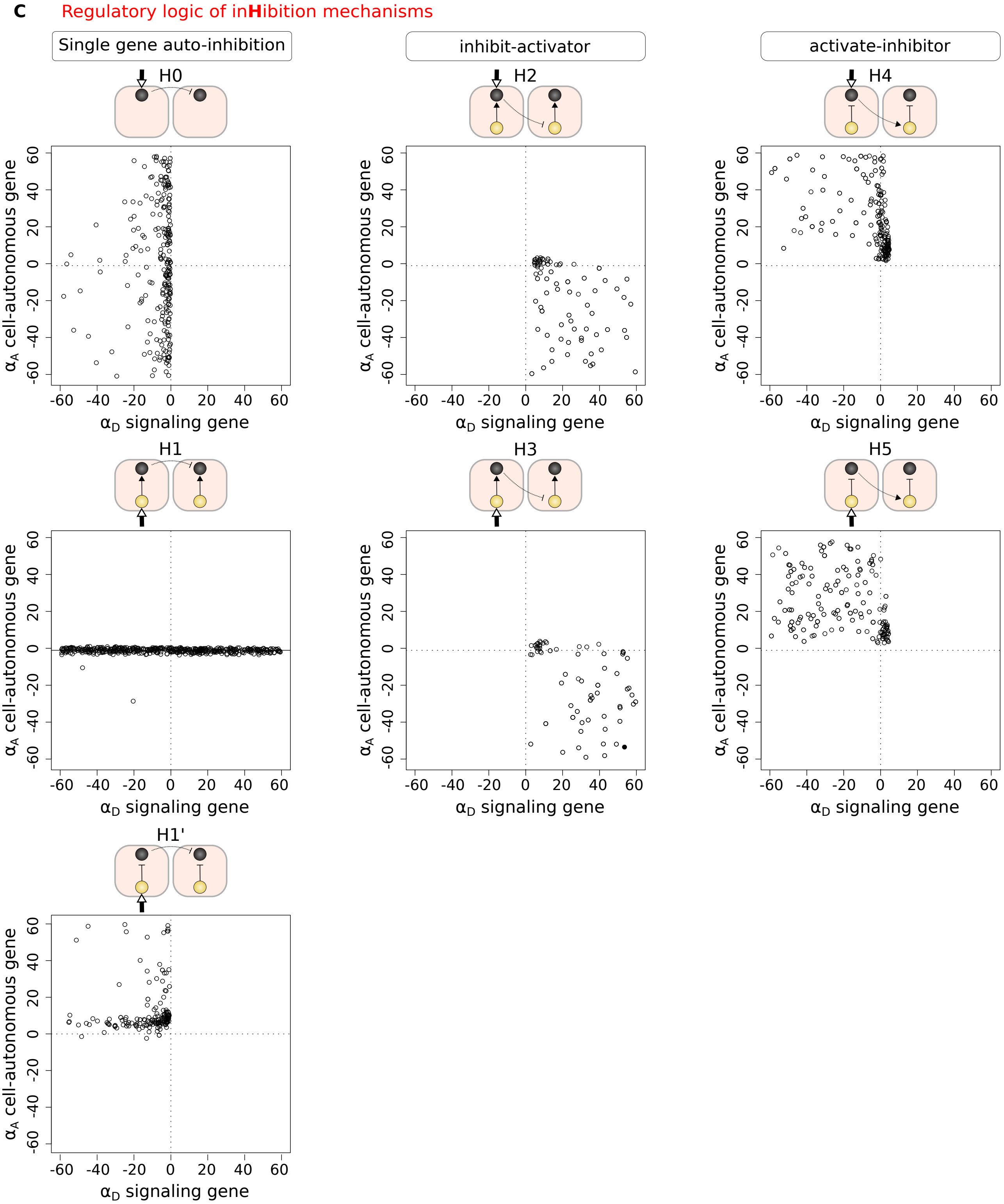

Appendix Figure S3: **Distinct mechanisms make use of distinct regulatory logics.** (A) Table of regulatory logic used by each minimal induction and inhibition circuit. We consider that when α belongs to a [-60:0] range, a gene is constitutively expressed: the gene is transcribed in the absence of input or despite a negative input. When α belongs to [0:60] different amounts of total input are necessary for the gene to be expressed. Introducing α in our regulatory function is key to the finding of a high diversity of mechanisms. Indeed, each mechanism makes use of distinct regulatory logic. For example, mechanisms that hold negative interactions within their circuitry, such as *auto-inhibition* (H0-H1-H1') or *inhibit-inhibitor* (D4), tend to necessitate constitutive expression of one or both genes to achieve the pattern. (B-C) For every parameter set of each minimal circuit we plot the corresponding values of α_A and α_D.

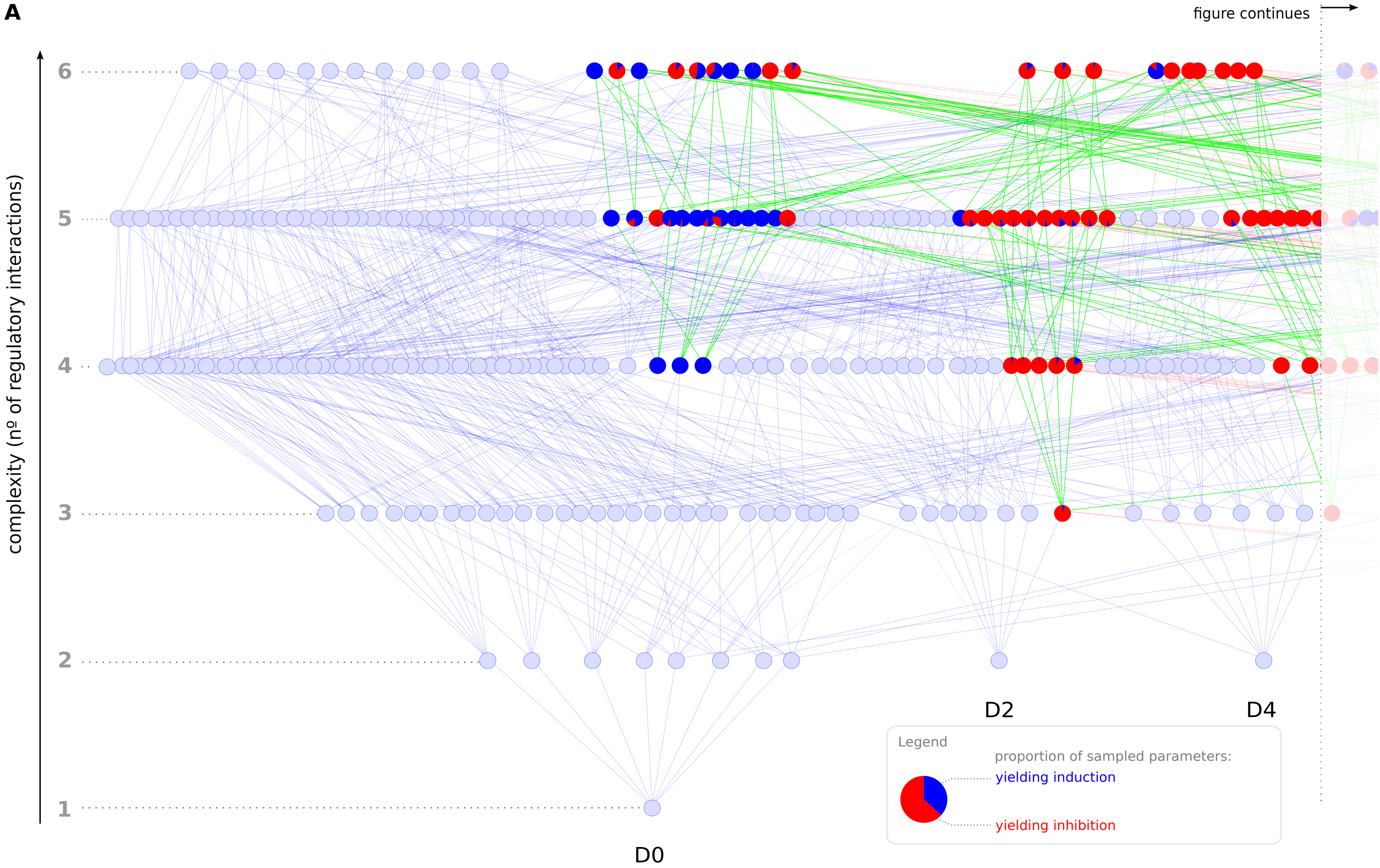
A
figure continues
complexity (nº of regulatory interactions)
6
5
4
3
2
1
D0
D2
D4
Legend
proportion of sampled parameters:
yielding induction
yielding inhibition

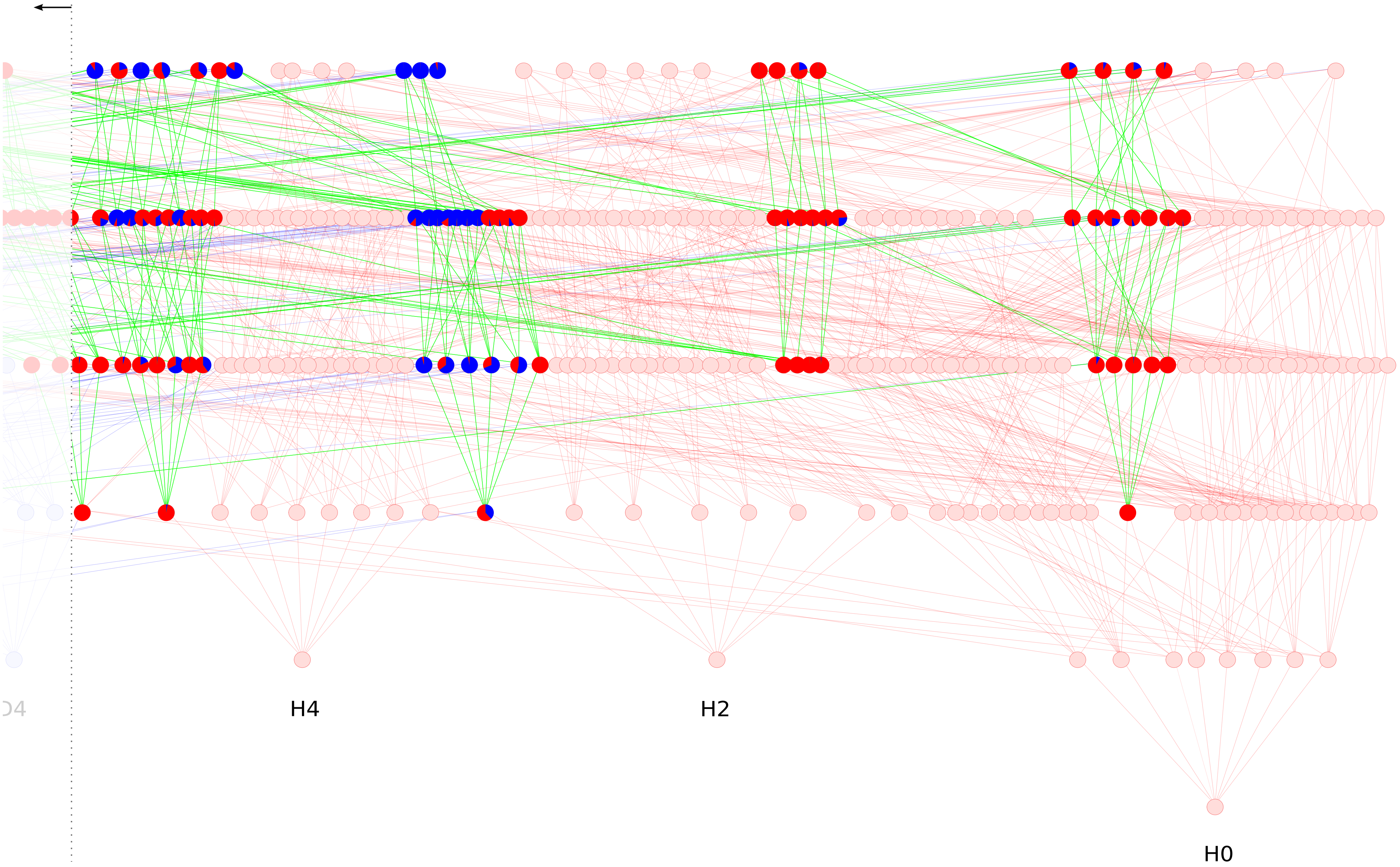
H4
H2
H0

B

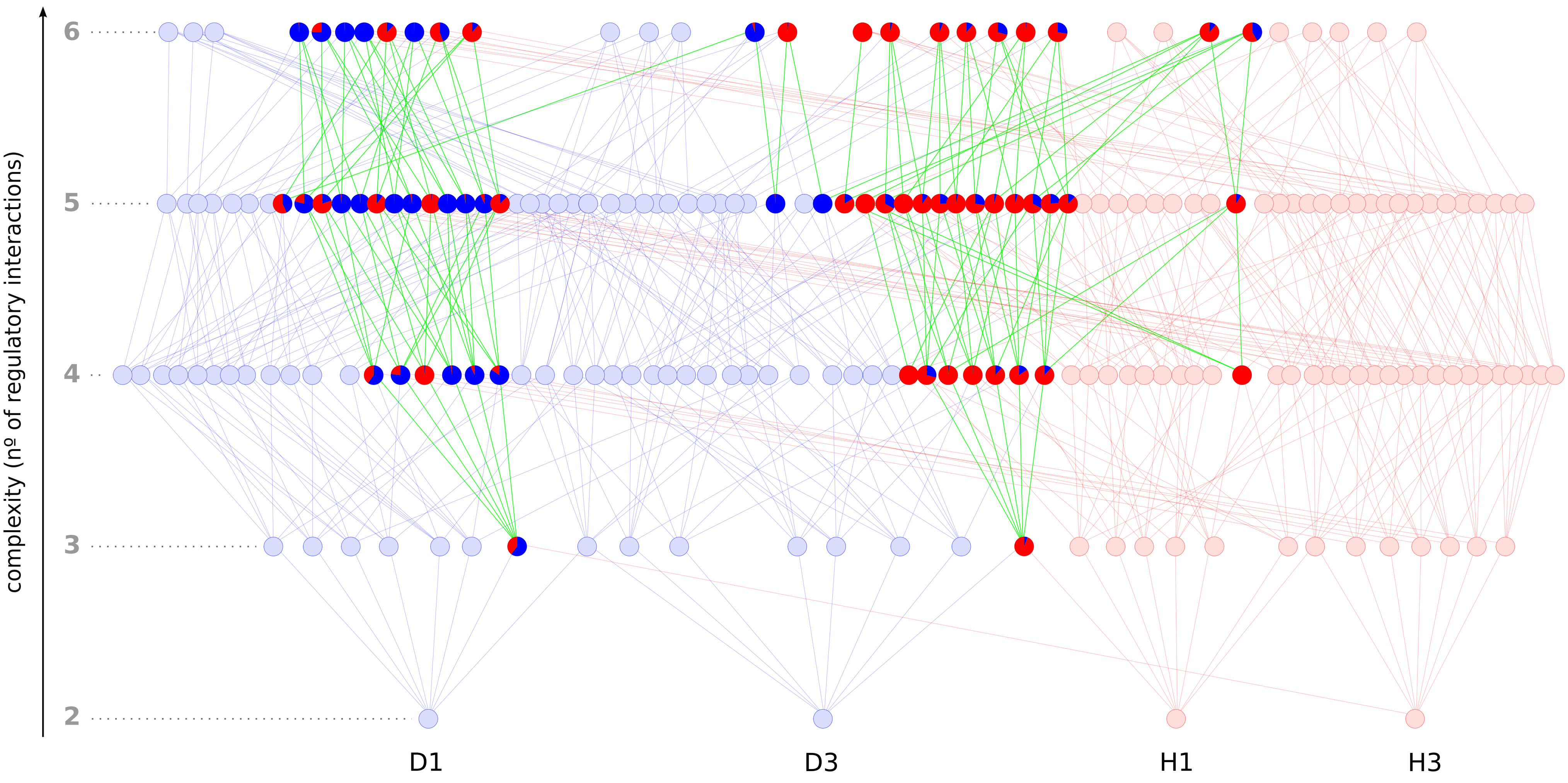

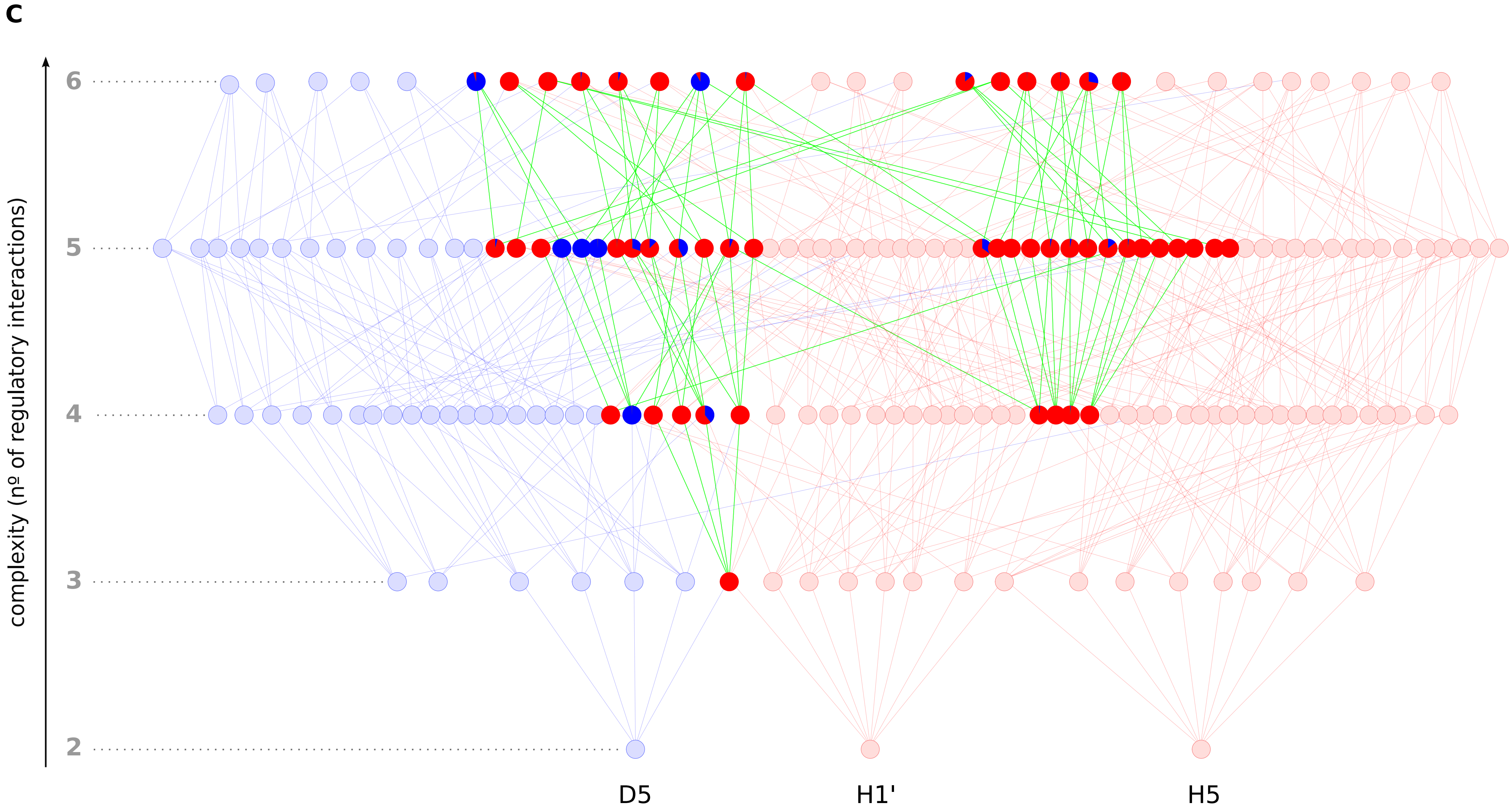

Appendix Figure S4: **Specialization of bi-functional topologies.** For each bi-functional topology (green nodes in Fig 3A) we show the proportion of the 10^7 sampled parameters that yield induction or inhibition by showing each node as a pie chart. We observe that most bi-functional topologies are strongly biased towards one of the functions (i.e. pie-charts which appear almost entirely blue or entirely red) and that the distribution of probabilities to achieve each function depends on the connectivity of a particular topology in the atlas. For example, topologies biased towards induction tend to connect to lower-complexity induction mono-functional topologies.

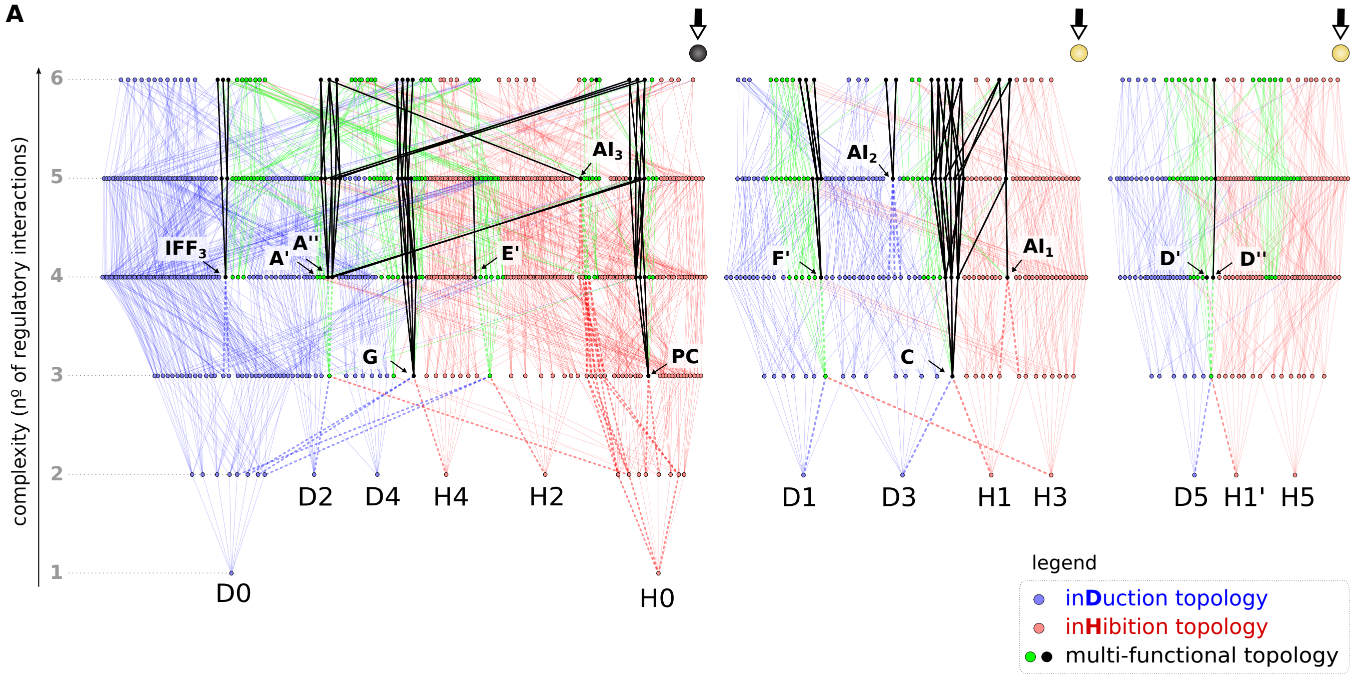

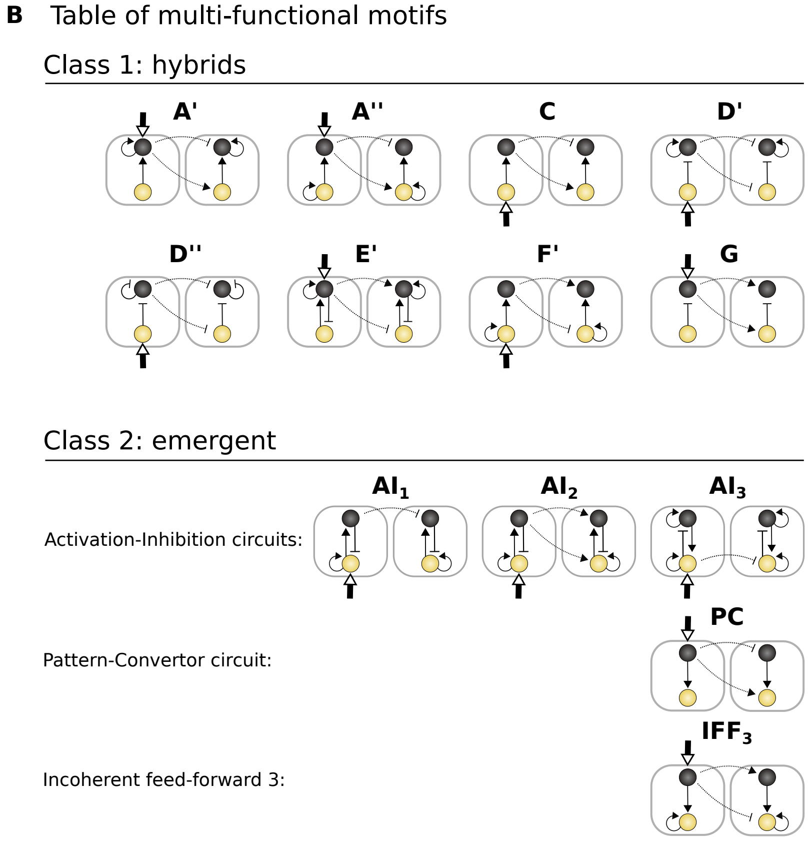

C Robustness of multi-functional circuits

Class 1: hybrids

A'	63	D''	2
A''	56.336	E'	1
C	7	F'	8
D'	1	G	1

Class 2: emergent

AI_1	10
AI_2	35
AI_3	2
PC	2
IFF_3	1

parameter robustness measured as number of multi-functional parameter-sets out of 10^7 sampled

Appendix Figure S5: **Complete atlas of multi-functional gene circuits.** (A) Complexity atlas showing (in black) the 72 topologies able to switch between induction and inhibition depending on the tissue context. The black stalactites help us identify the 13 minimal core multi-functional motifs. (B) Table of multi-functional motifs classified into two distinct classes: *hybrid* and *emergent*. (C) Robustness of the 13 minimal multi-functional motifs is measured as parameter robustness, i.e. the number of successful multi-functional parameter-sets out of 10^7 sampled.

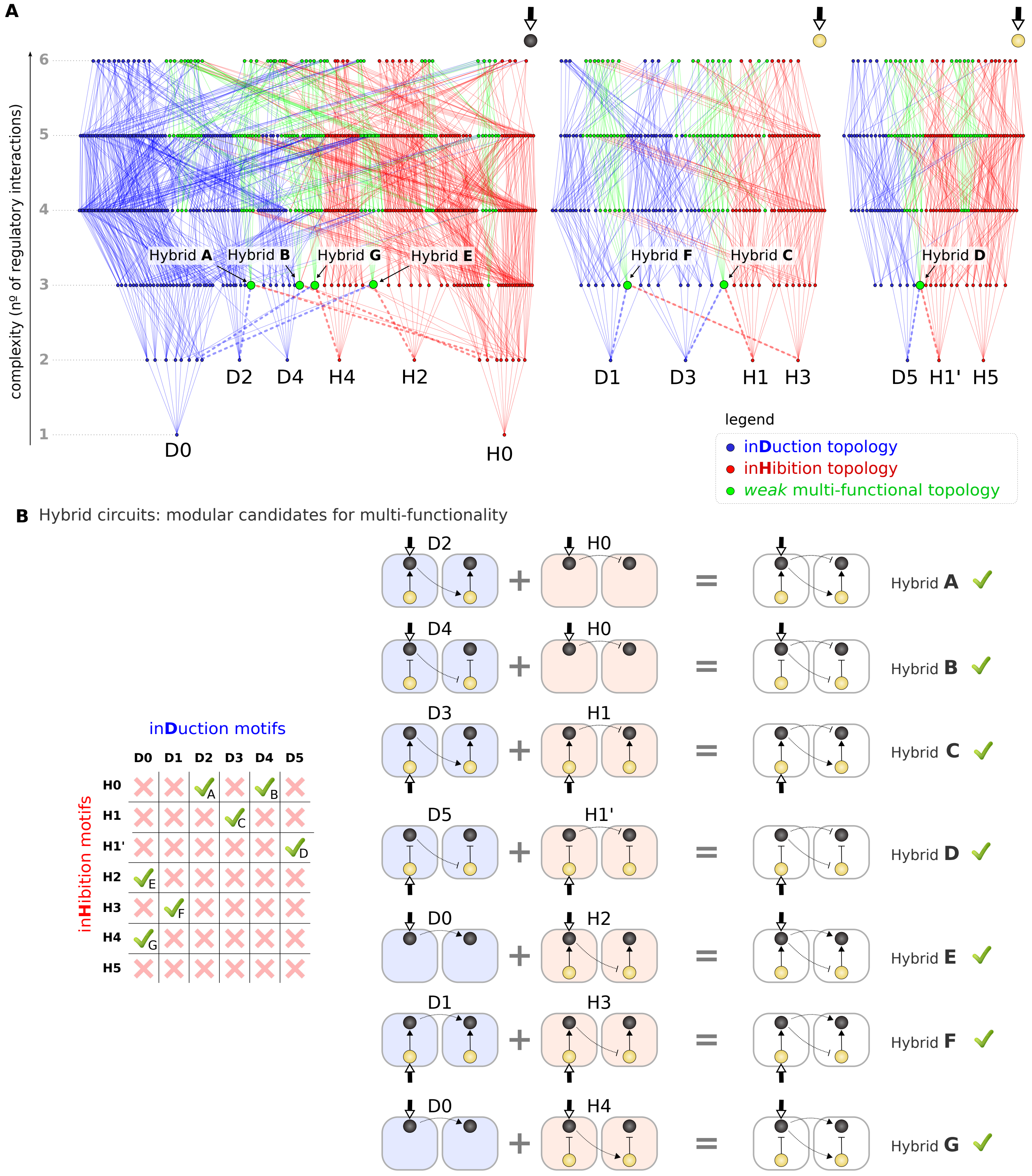

Appendix Figure S6: **Modular candidates for multi-functionality.** (A) *Hybrid* circuits are found connected to two lower-complexity induction and inhibition core circuits. (B) *Hybrid* circuits are the compatible union between a core induction and inhibition circuits.

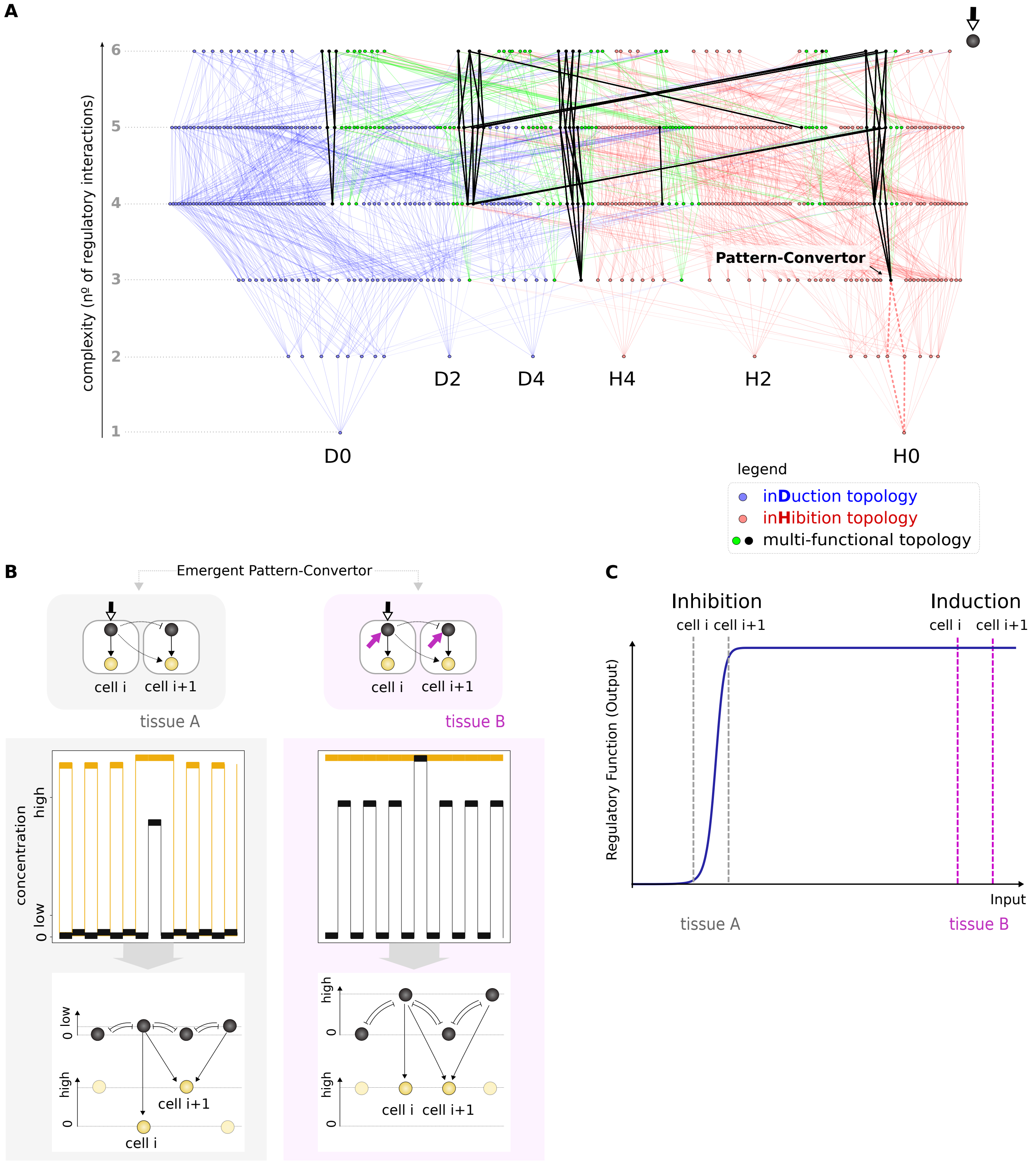

Appendix Figure S7: **Function-switching mechanism of Pattern-Convertor.** (A-B) We discuss this mechanism following a simple toy model. The auto-inhibition core circuit found within the circuit's architecture -H0- leads to a lateral inhibition pattern for the signaling gene (black) in both tissues. However, when the context signal C is present, the amplitude of the pattern (difference in concentrations of the signaling gene for two consecutive cells) is higher. The cell-autonomous gene (yellow) functions as a *convertor* as it feeds from the signaling gene (black). Each cell-autonomous gene reads-out three positive inputs from the signaling gene: two from neighboring cells and one from the same cell. Because the signaling gene holds an alternating pattern, the sum of these inputs on the cell-autonomous gene differs between two consecutive cells. The cell-autonomous gene converts the low-amplitude inhibition pattern of the signaling gene into an inhibition pattern and the high-amplitude inhibition pattern of the signaling gene into an induction one. (C) Inputs received by the cell-autonomous gene in two consecutive cells depending on the tissue. The shape of the regulatory function allows the following switching-mechanism: in tissue A (C=0) the input received by cell_i is sufficient to activate the cell-autonomous gene in that cell, while in its neighbor $\text{cell}_{\text{i}+1}$ it is not (inhibition); instead in tissue B (C=1) both cells receive sufficient input to be activated (induction).

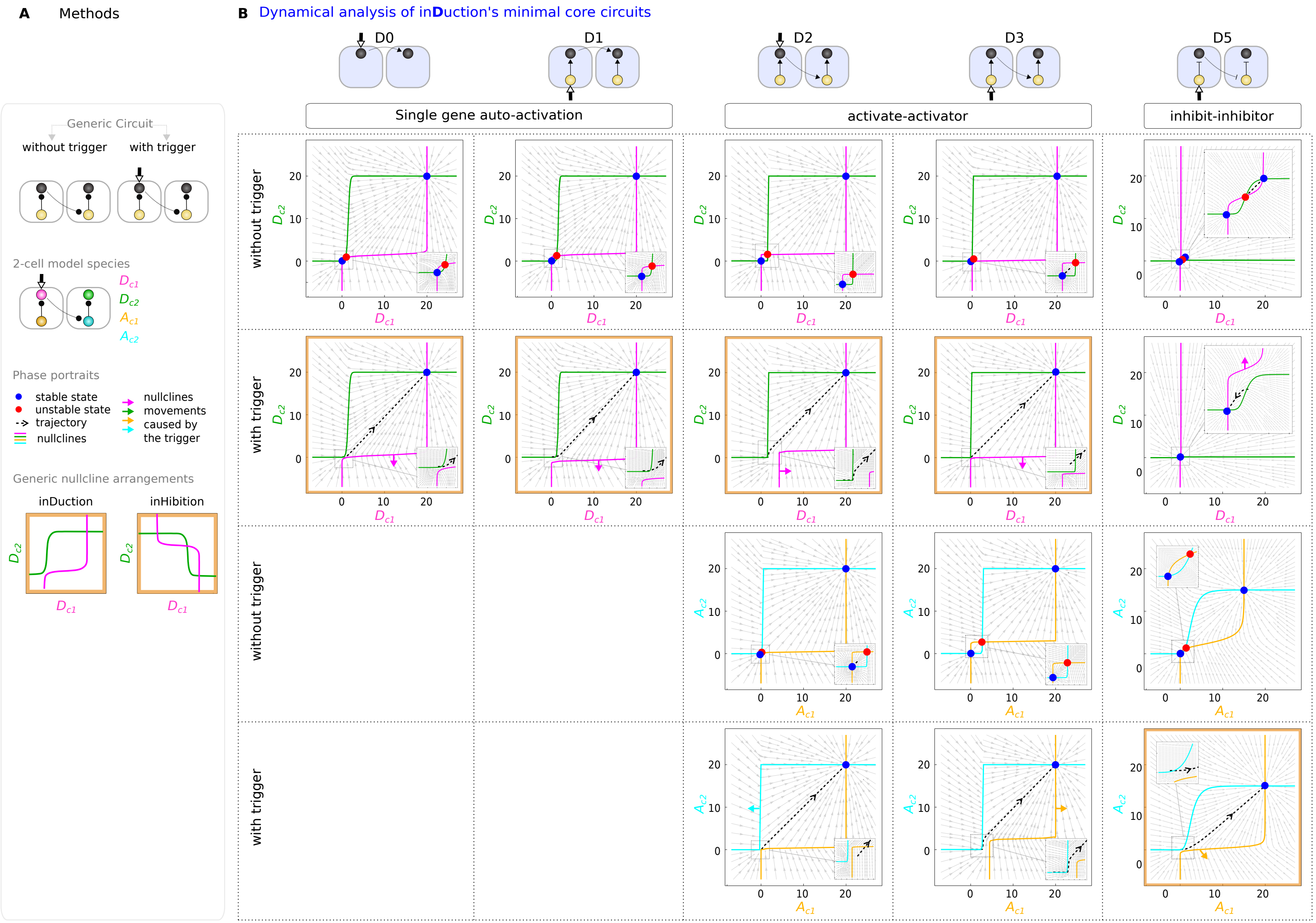

A
Methods
B
Dynamical analysis of inDuction's minimal core circuits
D0
D1
D2
D3
D5
Single gene auto-activation
activate-activator
inhibit-inhibitor
Generic Circuit
without trigger
with trigger
2-cell model species
D_{c1}
D_{c2}
A_{c1}
A_{c2}
Phase portraits
stable state
unstable state
trajectory
nullclines
nullclines movements caused by the trigger
Generic nullcline arrangements
inDuction
inHibition
without trigger
with trigger
without trigger
with trigger

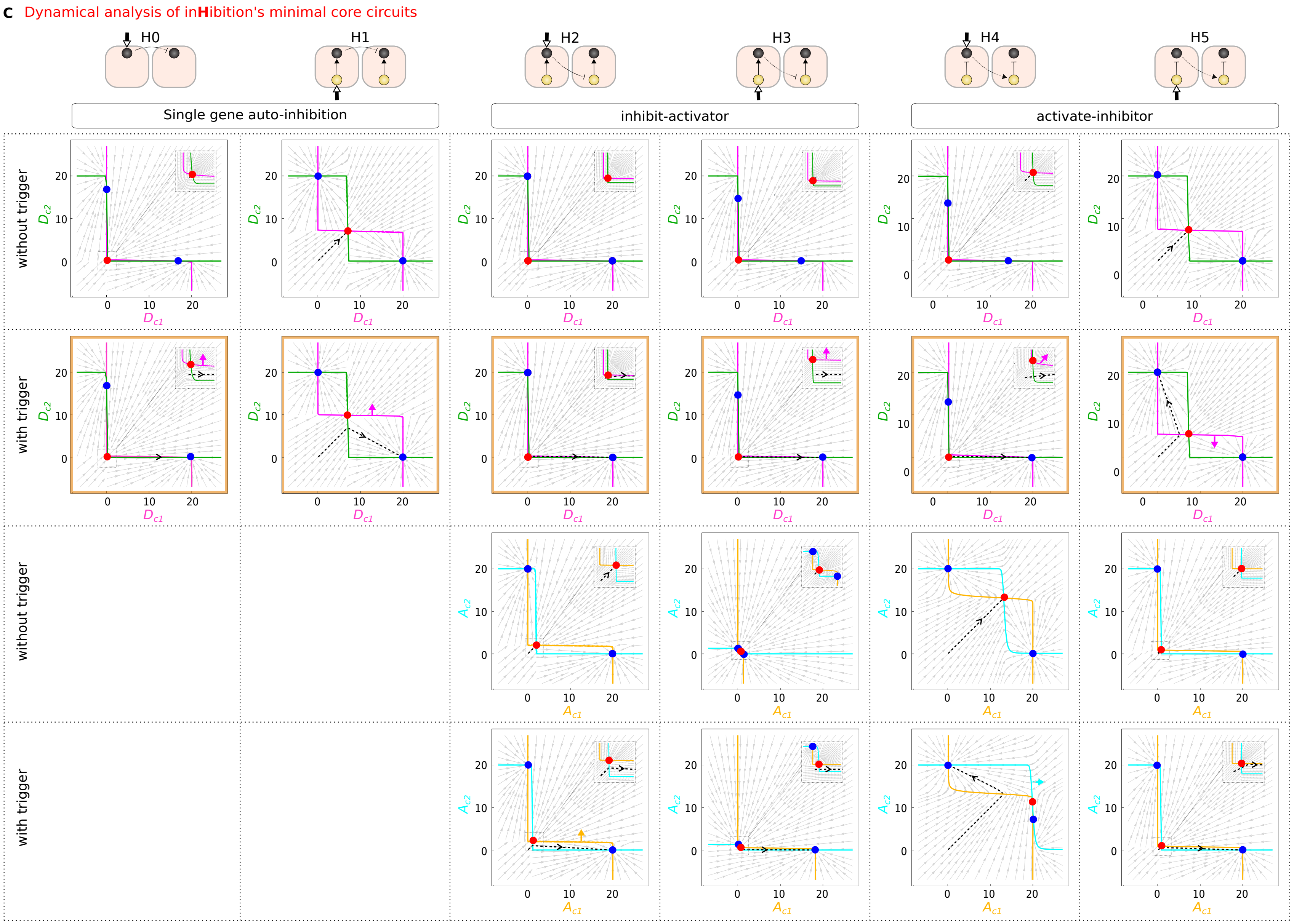

Appendix Figure S8: **Phase portrait analysis of induction and inhibition minimal circuits.** (A) In order to follow how the concentration of each of the four species of a circuit evolve we provide D_{c1}/D_{c2} and A_{c1}/A_{c2} phase portraits. (B) The annihilation that causes the system to shift to a [high-high] inductive state is characteristic of all minimal induction circuits, which share a general arrangement of their phase portraits. (C) The arrangement of nullclines and steady-states (attractors at [high-low] and [low-high], and an unstable steady state in between) is identical for all minimal inhibition circuits.

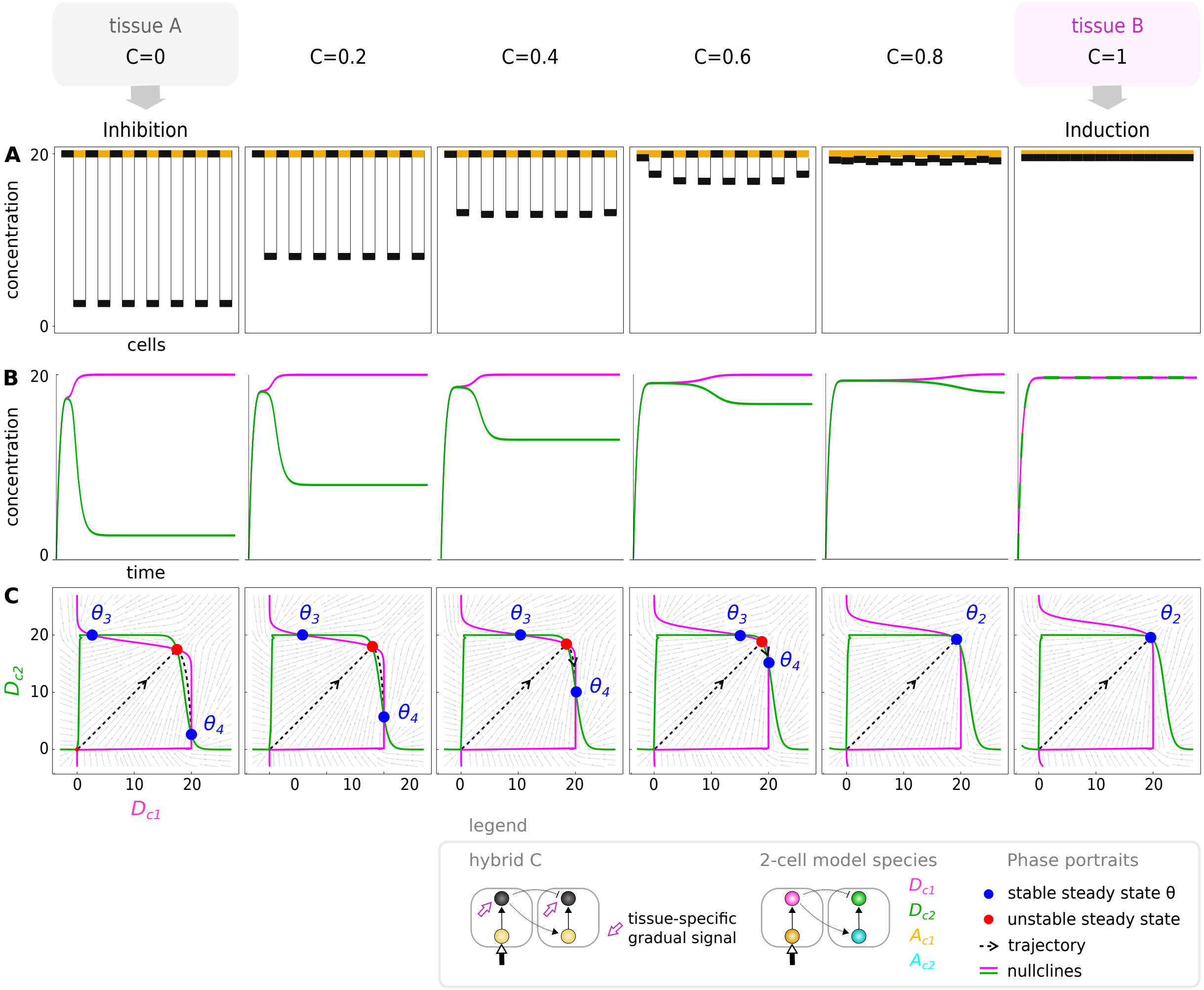

Appendix Figure S9: **Bifurcation as a function-switching mechanism.** We chose to explore hybrid C and follow how concentrations of the species D_{c1} and D_{c2} evolve as the context signal C gradually adopts distinct values from 0 to 1. We follow the switch from inhibition to induction in 3 panel rows which represent respectively (A) the final multicellular pattern (B) concentrations of $D_{c1}(t)$ and $D_{c2}(t)$ and (C) the corresponding phase portraits. The context behaves as a bifurcation parameter leading a *supercritical pitchfork bifurcation* [3]. In this type of bifurcation two stables states move towards each other, later collide and mutually annihilate to create a new stable state. This way, from an initial [high D_{c1} - low D_{c2}](attractor θ_4) inhibition state in tissue A, the system follows the moving attractor as D_{c1} is kept high and D_{c2} increases to finally transition to a [high D_{c1} -high D_{c2}] (attractor θ_2) induction state. This way, an inhibition pattern gradually reduces its amplitude to continuously transition to induction.

References

[1] Omer Barad, Eran Hornstein, and Naama Barkai. Robust selection of sensory organ precursors by the notch-delta pathway. *Curr Opin Cell Biol*, 23(6):663–667, Dec 2011.

[2] J. R. Collier, N. A. Monk, P. K. Maini, and J. H. Lewis. Pattern formation by lateral inhibition with feedback: a mathematical model of delta-notch intercellular signalling. *J Theor Biol*, 183(4):429–446, Dec 1996.

[3] Steven H Strogatz. *Nonlinear dynamics and chaos: with applications to physics, biology, chemistry, and engineering*. Westview press, 2014.
