# Supplementary material for: A spectrum of modularity in multi‐functional gene circuits
Source: Mol Syst Biol. 2017 Apr 1;13(4):925. doi: 10.15252/msb.20167347 (PMC5408781; doi:10.15252/msb.20167347)
Supplement: Supplementary file 2 — Movie EV1 [file MSB-13-925-s002.zip › MovieEV1/Legend_Movie_EV1.rtf]

Movie EV1. This movie shows how the instantaneous phase portrait, for hybrid C, changes as the context parameter C is gradually increased from C=0 to C=1. The 2 axes represent the levels of the gene D in 2 adjacent cells. The movie starts with the circuit acting as a lateral inhibition system (with 2 stable attractors (blue dots) at high-low and low-high), and ends as a lateral induction system (with a single attractor at high-high). See Figure 6 for more details.
